# The Awareness Among Parents About Cotton Earbud (Q-tips) Use in Children in the Jazan Region, Saudi Arabia: A Cross-Sectional Study

**DOI:** 10.7759/cureus.43734

**Published:** 2023-08-18

**Authors:** Ali M Shawish, Alhassan H Hobani, Lamees A Zaalah, Rafeef A Hakami, Ghaidaa H Alharbi, Roaa M Alhazmi, Bassam H Hakami, Mohammed H Matari, Khalid A Majrashi, Abduallah A Mawkili

**Affiliations:** 1 College of Medicine, Jazan University, Jazan, SAU; 2 Department of Surgery, Jazan University, Jazan, SAU

**Keywords:** cotton buds, jazan region, ksa, q-tips, awareness

## Abstract

Background and objective

The waxy substance secreted in the ear canal of mammals, including humans, is known as ear wax; it is also known by the medical term cerumen. It protects against germs, fungi, and water, helps with cleansing and lubrication, hydrates, and protects the skin of the human ear canal. The purpose of using cotton earbuds (Q-tips) is to clean the ear auricle (external part), relieve itching, and remove any excess water among other things; however, many people have been mistakenly using them for cleaning deeper spots in the ear, leading to some serious complications. Cotton earbud misuse has been documented to be the most common cause of accidental penetrating trauma of the eardrum among children. In this study, we aimed to determine the level of awareness of parents about the use of Q-tips among children and its consequences in the Jazan region, Saudi Arabia.

Methods

This was a cross-sectional study involving 391 participants conducted from February 2023 to April 2023 in the Jazan region. While all parents in the Jazan region were eligible to be included in the study, only those who responded to our survey questionnaire were recruited. Since our research focused on parents, participants who were aged less than 18 years and those who had never married were excluded. A validated questionnaire tested for reliability was used in the study. The association between the level of awareness about Q-tips use and the sociodemographic characteristics of the parents was evaluated using the chi-squared test. A p-value ≤0.05 was considered statistically significant.

Results

Out of the 391 individuals included in the survey, two-thirds (67.5%) were male and 32.5% were female, and most of them were married (89.8%). Regarding the knowledge of cotton bud use among the participants, we found that 12.3% agreed and 34% strongly agreed that cotton buds should be used to clean the ears. Furthermore, 74.9% of the participants knew that cotton buds could cause ear infections, 80.8% knew that cotton buds could cause eardrum perforation, and 79.3% knew that cotton buds could push ear wax deeper into the ear. We found that 152 (57.6%) of the male and 91 (71.7%) of the female participants used cotton buds on their own (p=0.011). Moreover, 176 (66.7%) of the male participants thought that cotton buds can damage their child’s ear while 69 (54.3%) of the female participants thought the same (p=0.048). When the participants were asked if they thought that the use of cotton buds caused complications, 155 (58.7%) of the males and 55 (43.3%) of the females said yes (p=0.015).

Conclusions

The parents in the Jazan region had a relatively good level of knowledge about ear cleaning using cotton buds among children. Nevertheless, we found a poor level of awareness about using cotton buds as the primary tool for cleaning the ears. Of note, 62.7% of the respondents knew that cotton buds could damage the ear, and 55% of the respondents thought that the use of cotton buds causes complications. However, 62.1% of the respondents still reported using it, and 50.1% of the respondents erroneously believed that it is beneficial to clean the ears with cotton buds. Otolaryngologists have always warned the public regarding the use of Q-tips and the complications they can cause. However, people are still ignorant as well as careless about these facts.

## Introduction

The waxy substance secreted in human ears is known as ear wax, and it is also known by the medical terms cerumen. It provides protection against germs, fungi, and water, helps with cleansing, hydrates, and protects the skin of the human ear canal [1]. Self-cleaning your ears with cotton earbuds (Q-tips) is a frequent habit among many people, even though the risks associated with it are well known [2]. The external auditory canal (EAC) has an adequate self-cleaning system since the ear wax migrates out with the epithelium towards the pinna aided by movements of the jaw, and hence using cotton bud can push cerumen further back into the EAC, causing wax impaction [3]. The use of Q-tips can cause eardrum perforation, deafness, otitis externa, cotton bud retention, acute injury to the EAC, and several other severe side effects [4]. Cotton buds or cotton swabs are made by wrapping a small wad of cotton around one or both ends of short sticks, which are typically made of wood, rolled paper, or plastic. Q-tips are cheap and widely available in drug stores and supermarkets. They are used by both adults and children, either by themselves or with the help of parents [2].

The purpose of using Q-tips is to clean the ear auricle (external part), relieve itching, and remove any excess water among other things, but many people have been mistakenly using them for cleaning deeper spots in the ear, leading to some serious complications including infections. It is a common public perception that the ear needs to be manually cleaned by an external object such as Q-tips to improve one's hygiene. However, the regular use of cotton buds can actually cause harm by impacting the ear wax and may lead to perforation of the eardrum. Cotton bud misuse has been documented as the most common cause of accidental penetrating trauma of the eardrum among children [5].

In fact, the use of Q-tips is not only unnecessary but can also cause serious damage to the ear. Otolaryngologists have always warned the public regarding the use of Q-tips and the complications they can cause. However, people are still ignorant about these facts, and the prevalence of manual ear cleaning is reportedly high, even among children. Hence, parents have the responsibility to educate themselves on this matter and stop using it on their children’s ears as well as their own. Although a number of studies have highlighted this crucial issue, no research has been conducted in the southern part of the Kingdom of Saudi Arabia on this important subject. In light of this, the goal of this study was to assess the parents’ awareness and knowledge about the use of Q-tips and its potential adverse consequences in Jazan, Saudi Arabia.

## Materials and methods

Study design and participants

This was a cross-sectional study conducted from February 2023 to April 2023 in the Jazan region. Our study included 391 participants who responded to the survey questionnaire we distributed. All parents in the Jazan region were eligible for the study, but only those who responded were recruited. Those who were unable to respond to the questionnaire, those who lived outside the Jazan region, those who were aged below 18 years, and those who had never married were excluded.

Sample size and data collection

The sample size for this study was calculated using the Raosoft sample size calculator [6], and 377 participants were needed to reach a 95% confidence interval and a 5% margin of error. After obtaining informed consent, the participant’s data were collected through an online survey distributed via social platforms among parents in Jazan. The questionnaire was developed by Aldawsari et al. [7]. Their permission was obtained to use the validated version in this study. The information collected included demographic data and socioeconomic factors of the respondents involved. Knowledge, practices, and awareness regarding the use of Q-tips were assessed thoroughly.

Data analysis

Data were extracted into an Excel sheet and subsequently refined before being imported into IBM SPSS Statistics for Windows, version 25.0 (IBM Corp., Armonk, NY) for statistical analysis. Frequencies with percentages were used to summarize categorical data. The chi-square test was performed to determine the association between sociodemographic characteristics and awareness about the use of Q-tips. A p-value ≤0.05 was considered statistically significant.

## Results

Out of the 391 participants in the survey, two-thirds (67.5%) were male; 39.4% were aged between 18 and 25 years, and 22.8% were between 26 and 35 years. Furthermore, 97.2% of the participants were Saudi citizens, 79.8% reported having a university degree, and 89.8% were in a marital relationship during the study period (Table 1).

**Table 1:** Sociodemographic characteristics of the participants

Variables	Frequency	Percent
Gender		
Male	264	67.5
Female	127	32.5
Age group, years		
18-25	154	39.4
26-35	89	22.8
36-45	80	20.5
46 and above	68	17.4
Nationality		
Saudi	380	97.2
Non-Saudi	11	2.8
Level of education		
Below secondary	8	2.0
Secondary	71	18.2
University	312	79.8
Marital status		
Married	351	89.8
Widow/widower	17	4.3
Divorced	23	5.9
Residency		
Urban	195	49.9
Rural	196	50.1

The details of the knowledge about cotton bud use among the study participants are shown in Table 2.

**Table 2:** Assessment of attitudes and knowledge regarding child ear cleaning

Variables	Strongly disagree		Disagree		Natural		Agree		Strongly agree	
	N	%	N	%	N	%	N	%	N	%
Cotton buds should be used to clean the ears	39	10	85	21.7	86	22.0	48	12.3	133	34.0
A damp towel or flannel should be used to clean the ears	21	5.4	71	18.2	90	23.0	61	15.6	148	37.9
Only water should be used to clean the ears	35	9.0	112	28.6	120	30.7	29	7.4	95	24.3
It is best not to clean the ears	137	35.0	132	33.8	62	15.9	17	4.3	43	11.0
Cotton buds are effective in removing ear wax	47	12.0	82	21.0	95	24.3	38	9.7	129	33.0
Cotton buds can cause infection of the ear	7	1.8	24	6.1	67	17.1	131	33.5	162	41.4
Cotton buds can cause a perforation (hole) of the eardrum	2	0.5	16	4.1	57	14.6	152	38.9	164	41.9
Cotton buds can push ear wax deeper into the ear	4	1.0	23	5.9	54	13.8	167	42.7	143	36.6

With regard to the practices and attitudes regarding child ear cleaning, we found that 69.8% of the participants clean their child’s ear for hygiene, and 30.4% for wax removal. As for the tool used for ear cleaning, most participants (92.6%) reported using cotton buds. Furthermore, 47.8% of the participants reported using cotton buds for more than five years, 68% of them reported occasionally using cotton buds for ear cleaning, and 7.4% used them daily (Table 3).

**Table 3:** Parent practices and attitude regarding child ear cleaning

Variables	Frequency	Percent
Reason for ear cleaning of your child (n=391)		
Hygiene	273	69.8
Ear wax removal	119	30.4
Itchiness	68	17.4
Habit	42	10.7
Feels blocked	53	13.6
Irritation	21	5.4
Tools used for ear cleaning of your child (n=391)		
Cotton bud	362	92.6
Key	19	4.9
Matchstick	11	2.8
Feather	14	3.6
Duration of use of cotton buds (n=391)		
Never used	74	18.9
Less than five years	130	33.2
More than five years	187	47.8
Frequency of using cotton buds for ear cleaning (n=391)		
Never used	88	22.5
Use cotton buds occasionally	266	68.0
Use it daily	29	7.4
Use it twice daily	2	0.5
Use more than twice daily	6	1.5

Regarding the use of cotton buds, 243 (62.1%) respondents used cotton buds on their own. On asked if they thought the cotton bud can damage the ear, 245 (62.7%) of the respondents said yes, 68 (17.4%) said no, and 78 (19.9%) were not sure. One hundred and ninety-six (50.1%) respondents believed that they gain a benefit from cleaning the ears with a cotton bud, 90 (23%) said there is no benefit in using it, while 105 (26.9%) were not sure if there is any benefit; 215 (55%) of the respondents thought that the use of cotton bud causes complications, 66 (16.9%) thought that there would be no complications, and 110 (28.1%) were not sure. We found that 160 (40.9%) of the respondents who thought that the use of cotton buds caused complications reported that their child has experienced otitis externa, and 159 (40.7%) of them stated that their child experienced ear pain. Most of the participants obtained their information about the use of cotton buds from health talks at the hospital (n=135, 34.5%) and from friends or neighbors (n=123, 31.5%) (Table 4).

**Table 4:** Awareness about the use of cotton buds and its complications

Variables	Frequency	Percent
Uses cotton buds		
Yes	243	62.1
No	101	25.8
Not sure	47	12.0
Do you think that cotton buds can damage your child's ear?		
Yes	245	62.7
No	68	17.4
Not sure	78	19.9
Are there any benefits of using cotton buds?		
Yes	196	50.1
No	90	23.0
Not sure	105	26.9
Do you think that the use of cotton buds causes complications?		
Yes	210	53.7
No	71	18.2
Not sure	110	28.1
If your answer to the previous question is yes, which complication has your child experienced?		
Otitis externa	160	40.9
Pain	159	40.7
Bleeding	44	11.3
Tinnitus	67	17.1
From where do you get information on the use of cotton buds?		
Health talk at the hospital	135	34.5
Friends/neighbors	123	31.5
Media (radio/TV)	91	23.3
Publications/journals	42	10.7

With regard to the association between awareness among the participants and their demographic characteristics, we found that demographic factors do have a significant effect on awareness with respect to certain aspects. Regarding gender and the use of cotton buds, we found that 152 (57.6%) of the male and 91 (71.7%) of the female participants used cotton buds on their own (p=0.011). Moreover, 176 (66.7%) of the male participants thought that cotton buds can damage their child’s ear while 69 (54.3%) of the female participants thought the same (p=0.048). When the participants were asked if they thought that the use of cotton buds causes complications, 155 (58.7%) of the males and 55 (43.3%) of the females said yes (p=0.015). When the participants were asked if they thought that cotton buds can damage their child’s ear, 97 (63%) of those aged between 18-25 years, 66 (74.2%) of those aged between 26-35 years, 36 (45%) of those who aged between 36-45 years, and 46 (67.6%) of those aged 46 years and above said yes (p=0.001). When the participants were asked if they thought that the use of cotton buds causes complications, 80 (51.9%) of those aged between 18-25 years, 61 (68.5%) of those aged between 26-35 years, 39 (48.8%) of those aged between 36-45 years, and 30 (44.1%) of those aged 46 years and above said yes (p=0.003). Regarding the level of education and the use of cotton buds, we found that seven (87.5%) of the participants with education below the secondary level, 42 (59.2%) with a secondary level education, and 194 (62.2%) with a university level education used cotton buds on their own (p=0.049). Regarding the marital status and the use of cotton buds, we found that 217 (61.8%) of the married participants, eight (47.1%) of the widowed, and 18 (78.3%) of the divorced participants used cotton buds on their own (p<0.001). When the participants were asked if they thought that cotton buds can damage their child’s ear, 133 (68.2%) of those who lived in urban areas and 112 (57.1%) of those who lived in rural areas said yes (p=0.021) (Table 5).

**Table 5:** Association between sociodemographic characteristics and awareness about the use of cotton buds

Variables	Uses cotton buds	Do you think that cotton buds can damage your child’s ear?	Is there any benefit in using cotton buds?	Do you think that the use of cotton buds causes complications?
Gender, n (%)				
Male (n=264)	152 (57.6%)	176 (66.7%)	133 (50.4%)	155 (58.7%)
Female (n=127)	91 (71.7%)	69 (54.3%)	63 (49.6%)	55 (43.3%)
P-value	0.011	0.048	0.886	0.015
Age group, years, n (%)				
18-25 (n=154)	97 (63.0%)	97 (63.0%)	85 (55.2%)	80 (51.9%)
26-35 (n=89)	50 (56.2%)	66 (74.2%)	45 (50.6%)	61 (68.5%)
36-45 (n=80)	56 (70.0)	36 (45.0%)	41 (51.2%)	39 (48.8%)
46 and above (n=68)	40 (58.8%)	46 (67.6%)	25 (36.8%)	30 (44.1%)
P-value	0.606	0.001	0.221	0.003
Nationality, n (%)				
Saudi (n=380)	237 (62.4%)	238 (62.6%)	190 (50.0%)	206 (54.2%)
Non-Saudi (n=11)	6 (54.5%)	7 (63.6%)	6 (54.5%)	4 (36.4%)
P-value	0.001	0.988	0.923	0.259
Level of education, n (%)				
Below secondary (n=8)	7 (87.5%)	5 (62.5%)	4 (50.0%)	3 (37.5%)
Secondary (n=71)	42 (59.2%)	39 (54.9%)	32 (45.1%)	33 (46.5%)
University (n=312)	194 (62.2%)	201 (64.4%)	160 (51.3%)	174 (55.8%)
P-value	0.049	0.558	0.900	0.333
Marital status, n (%)				
Married (n=351)	217 (61.8%)	222 (63.2%)	177 (50.4%)	195 (55.6%)
Widow/widower (n=17)	8 (47.1%)	12 (70.6%)	5 (29.4%)	6 (35.3%)
Divorced (n=23)	18 (78.3%)	11 (47.8%)	14 (60.9%)	9 (39.1%)
P-value	<0.001	0.397	0.386	0.214
Residence				
Urban (n=195)	121 (62.1%)	133 (68.2%)	103 (52.8%)	106 (54.4%)
Rural (n=196)	122 (62.2%)	112 (57.1%)	93 (47.4%)	104 (53.1%)
P-value	0.868	0.021	0.516	0.653

## Discussion

The use of Q-tips for ear cleaning is widespread in Saudi Arabia and other parts of the world [8-11]. The current study aimed to identify knowledge, practices, and awareness about ear cleaning with Q-tips among parents of children in the Jazan region. The analysis of the sociodemographic profile of the respondents showed that the majority were male, Saudi, married, and had a university degree. In addition, the findings of this study suggest that the use of Q-tips for ear cleaning is prevalent in the Jazan region. Our findings are consistent with previous studies from Saudi Arabia and other countries. A previous study from Riyadh reported that a significant proportion (42%) of the participants agreed or strongly agreed that Q-tips can be used to clean the ears [12]. In Nigeria, a study revealed that Q-tips are the most frequently used object to clean the ears [11]. Furthermore, in the present study, most of the participants (92.6%) reported using Q-tips as a tool for ear cleaning. The prevalence rate of cotton bud use in our study is in line with those reported in three studies conducted in the Sokoto metropolis (91.2%) [13], Corps Camp (93.4%) [14], and Aminu Kano Hospital (76.3%) [15]. However, Alrajhi et al. reported that Q-tips were used in Riyadh among 69.6% of both sexes: 53% in men and 47% in women [3]. Alshehri et al. discovered that Q-tips were the most commonly used instrument (77.7%) in Abha [1].

Regarding knowledge about ear cleaning, most participants disagreed with introducing objects into the ear canal. However, a sizable proportion still agreed about using Q-tips, damp towels, or water to clean ears. This highlights the existence of misconceptions about ear hygiene that need to be addressed through awareness campaigns. Most participants were also aware of the potential damage that Q-tips can cause, such as ear infections, eardrum perforation, and pushing ear wax into the ear. However, their knowledge is mixed with some misconceptions. In the current study, 74.9% understood that Q-tips could cause ear infections, 80.8% knew they could perforate the eardrum, and 79.3% knew they could push the ear wax deeper. Furthermore, 53.7% believed that the use of Q-tips causes complications, with the majority of the complications being otitis externa (40.9%) and ear pain (40.7%). These findings are consistent with the study conducted in Riyadh that revealed that the majority of the participants (75.8%) knew that Q-tips could cause ear infections; 78.9% knew that Q-tips could cause perforation of the eardrum, and 85.6% knew that Q-tips could push wax deeper into the ear [12]. Interestingly, in previous studies, only 18% of the respondents reported complications with Q-tips, and the most common complications reported were wax impaction (41.2%) and ear pain (39.7%) [3]; in a study at Jos University Teaching Hospital, 9.3% reported experiencing complications [16]. Foreign body invasion (40.7%) and ear canal damage (24.6%) were the most prevalent adverse events in a study conducted in Nigeria [15]. Another study found that 70.1% of participants had external otitis due to Q-tip use [17]. The correct purpose of putting Q-tips on the ears is to clean the auricle exclusively. However, their widespread misuse has led to many ear complications [18].

The current study also found that most participants were aware of the risks associated with using Q-tips, such as ear infections, eardrum perforation, and pushing the ear wax deeper into the ear. However, many participants still believed that Q-tips should be used for ear cleaning. A previous study revealed that Q-tips can cause cerumen impaction, damage to the ear canal, and even perforation of the eardrum [11]. In our study, although 74.9% of the participants knew that Q-tips could cause ear infections and 80.8% knew that they could cause eardrum perforation, most participants (92.6%) reported using Q-tips to clean their child's ears. This discrepancy between knowledge and practice suggests that awareness of the potential risks associated with using Q-tips does not necessarily translate into changes in behavior. Past research has revealed that most participants (65%) did not report complications, 16.2% reported having pain due to self-cleaning, and 16% reported external otitis [12].

The assessment of parents' practices concerning child ear cleaning showed that good hygiene was the reason most of them cleaned the ears, followed by wax removal. Q-tips were the most commonly used tool by the vast majority. Nearly half of the participants also reported frequent and long-term use of Q-tips. This indicates that excessive and improper ear cleaning is prevalent and can cause ear damage and injury. Alternative hygienic practices need to be promoted to replace Q-tips use. Our finding, consistent with previous studies, showed that the most common reasons respondents cited for using Q-tips were to clean the ears (34.2%) and remove wax (29.7%) [3]. Another survey found that 54.7% used Q-tips to clean their ears [15]. Another research indicated that hygiene was the main factor in self-cleaning (45%) [1]. On the contrary, in a study from Nigeria, 57% of the participants said that itching is the primary reason for using Q-tips; this could be due to the convenience of use and easy accessibility related to Q-tips in this community [11].

The study also found that demographic factors such as sex, age, education level, marital status, and area of residence significantly affected participants' awareness of the risks associated with cotton bud use. For instance, male participants were more likely to use Q-tips on their own, think that Q-tips can damage their child's ears, and believe that Q-tip usage causes complications compared to female participants. Married participants were more likely to use Q-tips for ear cleaning. Younger participants were more aware of the risks associated with using Q-tips, while those with a lower level of education were less likely to use Q-tips alone. Previous research showed a strong association between self-ear cleaning and gender, Q-tip ownership, and the belief that self-ear cleaning is beneficial [11]. However, in terms of the awareness of the adverse impact of using Q-tips, most participants were aware of the potential damage they caused and the complications that their children may have. However, nearly half also believed that there are benefits to cleaning ears with Q-tips. Therefore, this mixed awareness needs to be addressed through raising awareness and health education.

The primary sources of information on Q-tips in the current study were health talks at the hospital and friends and neighbors. The sources providing minor information were media channels and publications or journals. This result contrasts with previous studies that found that the media platforms provided most of the information while general practitioners gave the least [3].

The study provides valuable information on caregivers' knowledge, practices, and awareness regarding ear cleaning and using Q-tips in children. Misconceptions and unsafe practices seem to prevail in the community, which requires targeted health education to promote ear hygiene and prevent harm to child health. Policy interventions may also be needed to regulate the availability and marketing of Q-tips. Alwassel et al. have reported low levels of knowledge and awareness of the risks associated with using Q-tips in a Saudi community [19]. Alshehri et al. found that 56% had an intermediate understanding of ear self-cleaning in Abha, while 12% had poor knowledge [1]. Oladeji et al. reported limited knowledge among Nigerian community members on the dangers of using Q-tips [11].

The results of this study underscore the need for targeted interventions and public health campaigns to promote safer ear-cleaning practices and dispel misconceptions about the use of Q-tips. Health professionals, particularly otolaryngologists, pediatricians, and primary care providers, have a crucial role in educating parents and caregivers about the potential risks of using Q-tips and advising on alternative, safer ear-cleaning methods. In general, we believe the study's findings will help develop and implement appropriate targeted interventions to improve ear hygiene practices in the community.

## Conclusions

The parents in the Jazan region had a relatively good level of knowledge about ear cleaning among children. Nevertheless, we found a poor level of awareness with regard to using cotton buds as the primary tool for cleaning the ears. Of note, 62.7% of the respondents knew that cotton buds could damage the ear, and 55% of the respondents thought that the use of cotton buds causes complications. However, 62.1% of the respondents still reported using it, and 50.1% of the respondents erroneously believed that there is a benefit in cleaning the ears with cotton buds. Otolaryngologists have always warned the public regarding the use of Q-tips and the complications they can cause. However, people are still ignorant as well as careless about these facts. Further studies including various sections of society are required to gain deeper insights into the topic. Moreover, authorities should implement programs to raise awareness about safe practices for cleaning ears.
